# Ten striking facts about agricultural input use in Sub-Saharan Africa

**DOI:** 10.1016/j.foodpol.2016.09.010

**Published:** 2017-02

**Authors:** Megan Sheahan, Christopher B. Barrett

**Affiliations:** a Precision Agriculture for Development, Pilot House, 2 Atlantic Avenue, Boston, MA 02110, USA; b Charles H. Dyson School of Applied Economics and Management, Cornell University, 210B Warren Hall, Ithaca, NY 14853, USA

**Keywords:** Improved seed, Fertilizer, Agro-chemical, Machinery, Irrigation, Sub-Saharan Africa

## Abstract

Conventional wisdom holds that Sub-Saharan African farmers use few modern inputs despite the fact that most poverty-reducing agricultural growth in the region is expected to come largely from expanded use of inputs that embody improved technologies, particularly improved seed, fertilizers and other agro-chemicals, machinery, and irrigation. Yet following several years of high food prices, concerted policy efforts to intensify fertilizer and hybrid seed use, and increased public and private investment in agriculture, how low is modern input use in Africa really? This article revisits Africa’s agricultural input landscape, exploiting the unique, recently collected, nationally representative, agriculturally intensive, and cross-country comparable Living Standard Measurement Study-Integrated Surveys on Agriculture (LSMS-ISA) covering six countries in the region (Ethiopia, Malawi, Niger, Nigeria, Tanzania, and Uganda). Using data from over 22,000 households and 62,000 agricultural plots, we offer ten potentially surprising facts about modern input use in Africa today.

## 1 Introduction

Much of the sustained agricultural growth necessary for economic transformation comes from expanded input use, especially of modern inputs—like improved seed, fertilizers and other agro-chemicals, machinery, and irrigation—that embody improved technologies. Asia and Latin America enjoyed tremendous increases in agricultural productivity in a relatively short period of time through rapid and widespread uptake of yield-enhancing modern agricultural inputs (Johnson et al., 2003). The gains from diffusion of these inputs were enjoyed broadly, including to consumers (Evenson and Gollin, 2003), helping to stimulate historically unprecedented economic growth and poverty reduction in east and southeast Asia (David and Otsuka, 1994). It is well-acknowledged that Sub-Saharan Africa (SSA) did not participate to the same degree in the Green Revolution of the 1970–80s and has, therefore, not been able to reap the economy-wide rewards associated with input use expansion. Indeed, low use of modern inputs is nearly synonymous with African agriculture and acts as a motivation for the policy priorities set forth in forums such as the Abuja Declaration, Malabo Declaration, and under the Comprehensive Africa Agriculture Development Programme (CAADP).

But has no progress been made in input use over the last several decades in SSA? Should the rhetoric surrounding modern agricultural input use promotion remain unchanged? There are many reasons to expect it may be time to check the current accuracy of existing wisdoms about the African agricultural input use landscape. Most obviously, several governments have recently reinstated or revitalized agricultural input subsidy schemes aimed at promoting access to chemical fertilizers and improved seeds (Minot and Benson, 2009), with variable success (Jayne and Rashid, 2013). Irrigation and mechanization technologies have received far less policy attention, potentially translating into stagnation or even the reversal of prior progress in expanding their use (Mrema et al., 2008, Van Koppen, 2003). Meanwhile, factors external to agricultural policy—such as record high international food prices, urbanization, rapid growth of a middle class, increased access to market and other information through cell phones, and transformation of some food marketing channels—may have changed on-farm incentives and resulted in updates to farm management practices, including modern input use (e.g., Reardon et al., 2009, Tiffen, 2003). Furthermore, increased awareness of climate change and soil erosion may also be influencing farmers’ practices related to inputs (Nelson et al., 2010).

Despite these changes in the policy and operating environment, many prevailing beliefs about input use remain rooted in ideas formed 10–20 years ago, before the onset of what seems to be an African agricultural renaissance. Most knowledge of modern input use is currently derived from macro-level statistics, which cannot capture within-country heterogeneity and are prone to issues of data reliability (Jerven, 2013), or from studies using small or purposively chosen samples, which may not be reliably scalable for informing national- or multinational-level policy priorities. In spite of myriad studies focusing on some specific facet of modern input use in SSA, our understanding of the current input landscape at the country and continent level remains inadequate for guiding the next generation of agricultural policies and investments in the region.

The nationally representative, recently collected, agriculturally intensive, and cross-country comparable data sets provided through the Living Standard Measurement Study-Integrated Surveys on Agriculture (LSMS-ISA) Initiative, inclusive of some of the most populous countries in Africa, offer the timely opportunity to provide a more up-to-date platform for informing policy related to a bundle of inputs used by farming households. These data sets allow us not only to compute national-level statistics derived from household responses about their input use, representing cross-checks against the country-level statistics derived from macro-data that often form the basis of conventional wisdom, but also to study within-country and even within-household variation in input use levels that may be important considerations for the policy formation process. Further, because the LSMS-ISA effort includes the collection of global positioning system (GPS) information related to households and plots, the abundance of data therein can also be linked to external and increasingly plentiful and rich geospatial data sets containing a range of relevant covariates.

In Sheahan and Barrett (2014) we utilized one cross section of LSMS-ISA data collected between 2010 and 2012 in each of six countries (Niger, Nigeria, Ethiopia, Malawi, Tanzania, Uganda), including over 22,000 cultivating households and 62,000 agricultural plots, to produce a large number of descriptive statistics related to a set of inputs often cited as “under-used” in SSA: fertilizer, improved seed varieties, agro-chemicals (pesticides, herbicides, and fungicides), irrigation, and animal power and mechanized farm equipment. In this synthesis article, we focus on ten key facts we found most striking and important to pushing forward today’s research and policy frontier related to agricultural input use. The ten “new” (or, in some cases, “newly verified”) facts that follow are founded purely on descriptive analysis; our aim is not to uncover the pathways and casual determinants of the conditions we describe. Instead, we focus on the more fundamental goal of getting the basic truths right, an essential and to-date-overlooked step in the intensifying debates about how to stimulate African agricultural development. While a multitude of other interesting and policy relevant correlates exist that expose the great degree of heterogeneity across the region, we focus on just ten salient facts to help propel along the broader literature and policy debate.

## 2 Sample selection and variable creation

In order to create reliable and cross-country comparable descriptive statistics to underpin new understandings about agricultural input use in SSA, a major effort was undertaken to identify the appropriate underlying sample selection and variable creation process. The sample used in our analysis includes all households that cultivated at least one agricultural plot in a recent wave of LSMS-ISA data in Ethiopia (2011/12), Malawi (2010/11), Niger (2011/12), Nigeria (2010/11), Tanzania (2010/11), and Uganda (2010/11). For those countries where two seasons of agricultural data are available (Malawi, Niger, Tanzania, Uganda) our analysis focuses on the main agricultural season. Because the surveys are nationally representative (apart from Ethiopia, which is representative of the rural and small town population only) and not necessarily representative of the farming population, the portion of the total sample that we use differs across countries.^1^ Then, since most input use is observed at the agricultural plot, not household, level and much can be said about the within-farm variation in input use, some of the statistics that follow will also be calculated at the plot level.^2^ Table 1 describes the sample size for each country used in this analysis. Across the six countries, our sample includes 22,565 cultivating households and 62,387 agricultural plots, which represents nearly three-quarters of all households in the full surveys and is overwhelmingly but not exclusively rural.

Great attention was paid to ensure that computed input variables and covariates are as comparable as possible across countries despite sometimes large differences in how questions were asked or what type of information was extracted from survey respondents. This involved standardizing data cleaning rules and, in some cases, making assumptions about how best to aggregate specific input types within broader categories (e.g., mechanized inputs).^3^ We “clean” the transformed input use per hectare (generally kg/ha) values using a “winsorizing” technique, replacing extreme outliers beyond the 99th percentile with the value observed at that percentile under the assumption that all extreme values are due to measurement error. In some countries, we observe unreasonably extreme values in inorganic fertilizer application rates below the 99th percentile, and therefore apply additional winsorizing by replacing total application rates over 700 kg/ha, nitrogen application rates above 200 kg/ha, and phosphorous application rates above 100 kg/ha with those values. In those cases where a continuous variable (e.g., application amount) follows a binary input use variable, we allow the continuous amount to confirm the binary entry, meaning missing or zero continuous values are always assumed to denote “non-users.”

Since some of the inputs in which we have interest are best compared per unit of cultivated land, particularly application rates and area under irrigation, we put considerable effort into standardizing land size measures both within and between countries. In all of these surveys, farmer-reported plot sizes are complemented with GPS-based measures of some plots for comparison. Given evidence that self-reported measures of land size may contain bias and cause the misrepresentation of key relationships (Carletto et al., 2013), we use multiple imputation to arrive at a full set of GPS-based plot sizes where self-reported values are used as an instrument following the methodology described by Palacios-Lopez and Djima (2014). This major advance allows us to overcome some of the deficiencies of statistics derived from other household surveys where respondent error is acknowledged as likely but unable to be detected or eliminated.

Geo-referenced data also allow us to link any number of geo-spatial data sets to our constructed input variables. In this analysis in particular, we utilize geovariables matched by staff at the World Bank to the following external datasets: World Clim (rainfall), NASA’s Shuttle Radar Topography Mission (elevation), FAO’s Harmonized World Soil Database (soil nutrient availability), NASA and Boston University’s MOD12Q2 Land Cover Dynamics Database (vegetative greenness), Harvest Choice (agro-ecological zones), USAID’s Famine Early Warning Systems Network (distance to markets), and Africa Infrastructure Country Diagnostic and Road Agency Formation Unit (distance to roads). In addition to these secondary data sources, we also utilize standardized household demographic information from the World Bank’s Comparative Living Standards Project (CLSP) and household consumption aggregates constructed from the LSMS-ISA data by individual World Bank country offices.

Despite a purposively chosen sample of main season cultivators, we apply household level sampling weights and account for the complex survey design to construct nationally representative statistics (or, in the case of Ethiopia, representative of rural areas and small towns only). Further, at the plot level we apply household level weights multiplied by the imputed plot size (in hectares) so as to not overweight the importance of very small units of cultivation. Community level data on input use and markets captured in accompanying surveys administered to focus groups within communities, infrequently used in this analysis, are linked to household level variables in order to apply household level weights. Apart from all of the aforementioned cleaning and weighting considerations, most of what follows relies on simple analysis of statistical associations.

## 3 Ten new or verified facts

The descriptive statistics from the augmented LSMS-ISA data suggest that input use across SSA is far more complex than stylized prevailing beliefs derived from often-quoted macro-scale statistics and (dated or statistically non-representative) numerical values. Indeed, we uncover a rich story of uneven input use in African agriculture. We summarize key descriptive results in ten important or surprising findings that may help to guide policy choices, to serve as an empirical check on conventional wisdom, and to motivate a new wave of research to further our understanding of the agricultural input landscape in SSA. In some cases, we include tables or figures to illustrate our broader findings; in others we simply summarize what is available in considerably more detail in Sheahan and Barrett (2014).

### 3.1 Modern input use may be relatively low in aggregate, but is not uniformly low across these six countries, especially for inorganic fertilizer and agro-chemicals

In an environment where initial soil productivity may be low or where crops are cultivated without the ability to leave plots fallow, replenishing soil nutrients is essential for the long term viability of agriculture (Henao and Baanante, 2006). Several researchers have estimated that over 50% of productivity gains experienced during the Green Revolution in Asia can be attributed to increased use of inorganic fertilizer alone (Hopper, 1993, Tomich et al., 1995), suggesting that SSA may need to follow suit. Using FAOSTAT data from 2009, Minot and Benson (2009) find that SSA households apply an average of 13 kg of inorganic fertilizer per hectare of cultivated land, a statistic that has proliferated and prompted considerable pressure within African governments to stimulate fertilizer use, perhaps most prominently within CAADP policy dialogues, and a rise or reinstatement of government input subsidy programs. While there has been some acknowledgement that heterogeneity in fertilizer use conditions exists across the region (e.g., Morris et al., 2007), the assumption that most SSA farmers are under-utilizing fertilizer guides most of our narrative on the topic.

Using the LSMS-ISA data, we find that fertilizer use is considerably more widespread than is often acknowledged. As shown in Table 2, average inorganic fertilizer use rates are well above the widely quoted 13 kg/ha statistic in 3 of 6 countries, with a simple six country average nutrient application rate of 26 kg/ha (equivalent to 57 kg/ha total fertilizer). Application rates are highest in Malawi and Nigeria, both with government input subsidy programs, and Ethiopia, where the government sets (and subsidizes) fertilizer prices but does not consider it a formal subsidy programme (Rashid et al., 2013).^4^ Furthermore, we find that 35% of cultivating households use inorganic fertilizer in any amount in the main growing season, including over three-quarters of all cultivating households in Malawi, half in Ethiopia, and around 40% in Nigeria. In short, inorganic fertilizer use is far more widespread than common assumptions about African smallholder agriculture posit. Of the six countries, the application rates we compile using the LSMS-ISA data match the current FAOSTAT macro-statistics reasonably closely in four countries.^5^

In addition to depleted soil nutrients, crop yields can also be suppressed by pests, diseases, and weeds. Actual losses to major crops from all of these types of “pests” are estimated to be about 30% of attainable yields as collected from sources around the world (Oerke and Dehne, 2004). In an effort to control these unfortunate agricultural realities, farmers can apply agro-chemicals in the form of pesticides, herbicides, fungicides, and insecticides. Gianessi and Williams (2011) contend that herbicide use, in particular, remains a major unexploited means of increasing yields and saving labor on SSA farms. For instance, in their global analysis, Zhang et al. (2011) found that only 3% of global pesticide consumption came from Africa, 2% from South Africa alone, leaving only 1% for the remainder of the continent. But most such analysis relies on official government estimates using outdated data. Given some household-level evidence showing a steady increase in pesticide use over time (Williamson et al., 2008) and findings that households source pesticides from unregulated and informal markets (Williamson, 2003), these oft-cited figures might dramatically understate pesticide and other agro-chemical use in SSA.

Table 3 presents overall agro-chemical use statistics taken from the LSMS-ISA household survey data. In general, the percent of cultivating households applying an agro-chemical in the main growing season appears higher than conventional wisdom holds, with over 16% applying to their fields in the main cultivating season. These percentages are even higher in Ethiopia and Nigeria, where agro-chemicals are used by 30–33% of cultivators, which are slightly above what is reported in other major studies from our literature review (Table 3). Further, the statistics we describe relate only to chemicals applied to crops on the field, not those also used in storage. Using the same LSMS-ISA data, Kaminski and Christiaensen (2014) find that 63% of maize growing households in Uganda, 49% in Tanzania, and 11% in Malawi used some form of spraying or smoking of their crops while in storage, suggesting that on-field usage does not exhaust the full set of possible chemicals used in African agriculture. Agro-chemical use is perhaps more widespread than is commonly recognized in the region.

These findings might usefully prompt further research, especially because it is known that some of the chemicals used on farm in SSA are banned in other countries due to their toxicity (Williamson et al., 2008). Prior research in Asia established prospective environmental and human health effects of agro-chemicals use (e.g., Antle and Pingali, 1994), which must be weighed against the potential productivity benefits of non-trivial agro-chemicals use in African agriculture.

### 3.2 The incidence of irrigation and mechanization, however, remains quite small

Using global data to study total factor productivity, Fuglie (2008) finds that irrigated land is twice as productive as rainfed land after controlling for other factors. In SSA specifically, Fuglie and Rada (2013) report that average yields on irrigated fields are 90% higher than on nearby rainfed fields. Further, Evenson et al. (1999) find that one of the key factors in agricultural productivity growth in Green Revolution India was public investments in irrigation. Because of its perceived importance for productivity enhancement, where economically viable, the lack of irrigation is often a starting point in the discussion of low input use in SSA. In one of the most recent and disaggregated looks at irrigation across the region, Svendsen et al. (2009) use AQUASTAT/FAO data to show that Sub-Saharan Africans withdraw about one-quarter as much water as the per capita global average. Similarly, Rosegrant et al. (2009) claim that less than 3.5% of all agricultural land in SSA is irrigated.

Table 4 displays the range of irrigation statistics we can tally from the LSMS-ISA data. Across the six countries, we find that about 5% of households use some form of irrigation in the main growing season, covering only about 2% of land under cultivation. While slightly higher than the AQUASTAT/FAO numbers, the same data used by Rosegrant et al. (2009), the estimates still show a very low incidence of irrigation across these countries. As with respect to inorganic fertilizer and agro-chemical use, great heterogeneity exists. Ethiopia and Niger have the highest percent of cultivating households with some form of irrigation in the main season with Malawi at the lowest end.^6^ Of course, because the LSMS-ISA household data do not include large-scale commercial farms run as firms in the sampling frames for the household surveys, these figures are likely somewhat downwardly biased as estimates of overall agricultural production in these countries, especially given the finding by Svendsen et al. (2009) that large-scale irrigation projects currently make up the most significant portion of irrigated land in SSA. But given the modest extent of corporate farming in SSA and limited community irrigation opportunities observed in the LSMS-ISA data, the core narrative of minimal levels of irrigation holds up in the most recent data.

Traditional agricultural practices in SSA rely on human power channeled through hoes, shovels, cutlasses, and other hand tools to bring new land under cultivation, prepare fields for planting, and harvest crops. Mechanized equipment or animal traction can be employed to increase the timeliness of field preparation and expand farm size, all while saving labor and increasing agricultural productivity, should the right conditions exist. While studies on tractor and animal draught power use are far less prevalent than those of the other inputs studied in this paper, the consensus appears to be that reliance on human power for agriculture is still hugely dominant and limits productivity increases (e.g., Sims and Zienzle, 2006). In an overview of the current state of mechanization in SSA using FAOSTAT/AGS data, Mrema (2011) finds that there were 2 tractors per 1000 ha of arable land in 1980 but only 1.3 in 2003, as compared to the more than doubling of tractor prevalence in Latin America and Asia over the same time frame. This echoes other findings of decreasing tractor use over time by Pingali (2007). Ashburner and Kienzle (2011) also show a decrease in mechanization in SSA, claiming that primary preparation carried out by hand tools is currently employed on 80% of land area, with draught animal technology (DAT) only at 15%, and the remaining 5% using tractors. This can be compared with reports based on 1998 data that 65% of land under cultivation was done by hand, 25% using DAT, and 10% using engine power (Clarke and Bishop, 2002).

Because mechanization is a process and may express itself through the utilization of different technologies across different cultivating environments (Pingali et al., 1987), the LSMS-ISA surveys can only provide a picture of current use or ownership of inputs associated with mechanization. Table 5 shows that tractor ownership at the household level remains quite low, with around 1% of households across all countries claiming possession. The incidence of tractor rental appears no more robust, with a similar percentage of households engaging in the tractor rental market. As a means of comparison with the FAO statistics, we estimate the number of tractors in the country, as aggregated across the full sample and weighted to a national level using the population weights. The estimates in Nigeria and Tanzania far exceed those reported by the FAO likely due (in part but not entirely) to differences in the year the data were obtained and the small sample size off of which national ownership rates are estimated. The Malawi numbers are virtually identical, despite the huge time variation, which may signal that tractor use in Malawi has stagnated, as hypothesized about SSA more generally. Across the six countries, about 32% of households own and 12% of households rent some type of farm equipment that could be used for mechanization. The ownership or rental of other mechanized farm implements apart from tractors, therefore, is far greater in all countries apart from Malawi; however, differences in included equipment type by survey likely contributes to some of the heterogeneity in percentages across countries (see footnote 3).

In only a limited number of surveys (Ethiopia, Niger, Nigeria) do we observe whether particular types of traction animals or equipment were used on individual plots in the main growing season. Nigeria and Ethiopia show signs that traction animals or mechanized inputs are used in addition to or in replacement of human labor, implying that mechanization levels are not necessarily as miniscule as simple ownership statistics suggest. In Nigeria we observe that 27% of cultivating households used animal traction on their plots while 25% used machines on their plots, where 47% of households use one or the other. Because both of these values far exceed the percent of households owning traction animals and mechanized equipment, this suggests that the rental market for both is fairly substantial in Nigeria, where the government has dedicated significant resources to the promotion of agricultural mechanization in recent years (Takeshima et al., 2013). In Ethiopia, households were asked about the number of oxen they used to plow their fields. 43% of cultivating households claimed to use no oxen, 22% claimed to use only one, 27% claimed to use two, and 8% used more than two. Of the 65% of households with one or no oxen, about 30% said they instead applied manual labor to their fields with the remaining 70% saying they rented or borrowed another ox or used a different animal for plowing. In Niger, we observe community tractor access in the community level surveys. When matching those variables to the household level, we find about 9% of households live in communities where a tractor is available, but that only 0.2% of households claim to rent a tractor. Community tractor access in Niger appears either not to provide attractive mechanization opportunities or, alternatively, is not considered by survey respondents as part of the rental market. Overall, ownership of agricultural machinery remains rare among African farmers but much remains to be learned about rental and sharing arrangements that might enhance access for those who do not own equipment or draught animals.

### 3.3 Considerable variation exists within countries in the prevalence of input use and of input use intensity conditional on input use

Within-country input use patterns vary strikingly, a fact necessarily masked by macro-level statistics of the sort that commonly inform discussions of African agriculture. The LSMS-ISA survey data allow us to disaggregate input use patterns to reveal a great deal of heterogeneity across sub-national regions, agro-ecological zones, underlying soil types, as well as accordingly to the characteristics of individual households and plots. Beyond the characteristics that we chose to investigate in our analysis, there are near infinite potential sources of variation in input use that may be important to the substantial variation we observe. Notably, even more so than the agro-ecological zone distinction that can be geo-referenced to the household level data, we find that regional variation (i.e., across administrative boundaries) in input use within countries is immense, likely due to factors like input and output prices, market access, and past investments in infrastructure, agricultural extension services, etc.

In certain countries, particularly Ethiopia, this level of disaggregation illuminates the tremendous heterogeneity in input use across regions. Three regions (Tigray, SNNP, Harari) far surpass the national average unconditional total fertilizer application rate of 45 kg/ha, while five regions fall well below even 10 kg/ha (Afar, Somali, Benshagul Gumuz, Gambela, Dire Dawa). This large spread is also evident for other inputs, with ranges from 0 to 50% of households using agro-chemicals by region (relative to a national average of 31%) and 0–47% of households using irrigation (relative to 9% nationwide). In relatively lower input countries, like Niger and Uganda, we also find some sub-national variation and patches where input use is far greater than national averages suggest. In Niger, those regions with fewer cultivating households (Agadez, Diffa, Niamey) have relatively higher input use levels than the more prominent agricultural areas. However, even Dosso has two times the proportion of inorganic fertilizer users relative to the national average. In Uganda, the largest amount of within-country variation is observed in agro-chemical use, with the major agricultural areas having lower percentages of users than the minor cultivating areas, likely due to sample selection and size.

**Fig. 1:**
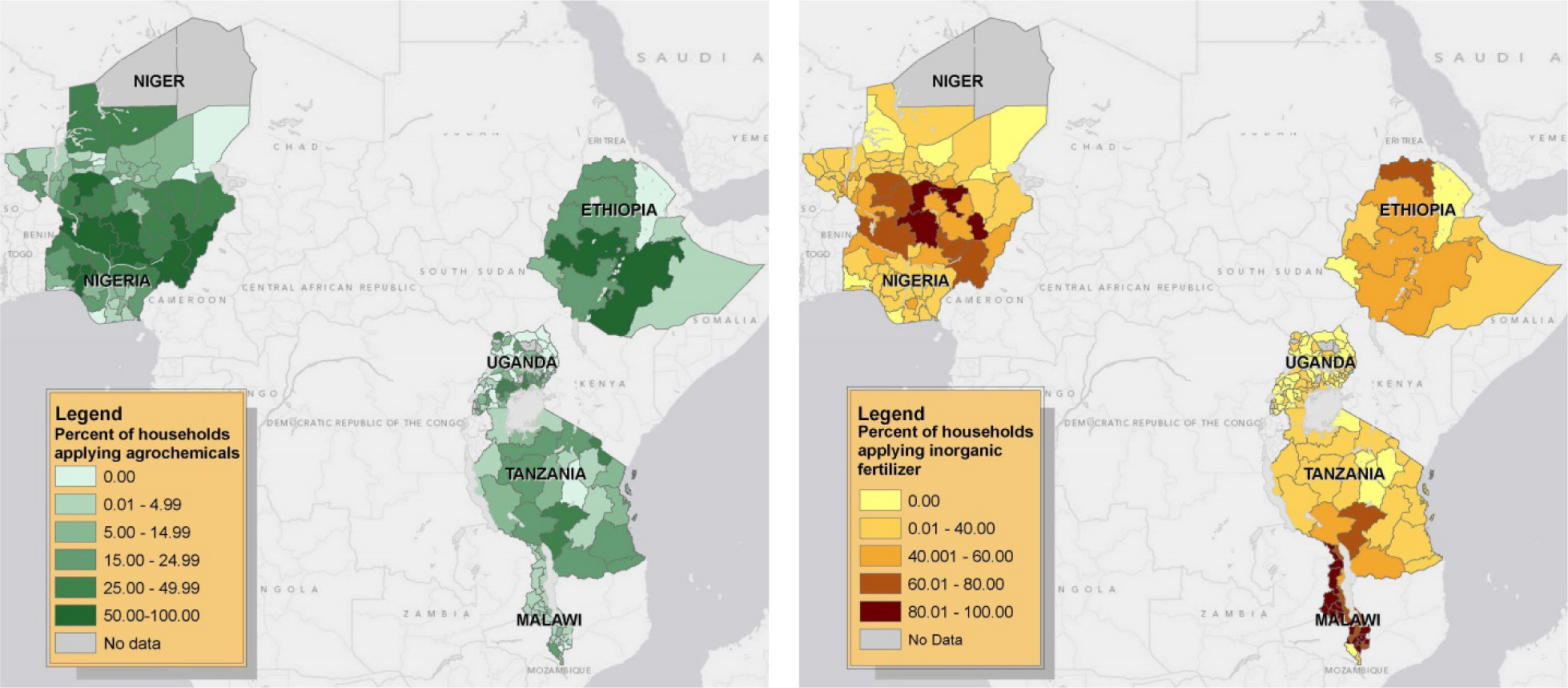
Agro-chemical (left) and inorganic fertilizer (right) use within LSMS-ISA countries.

*Source:* Authors’ calculations using the Living Standards Measurement Study Integrated Surveys on Agriculture: Ethiopia 2011/12, Malawi 2010/11, Niger 2011/12, Nigeria 2010/11, Tanzania 2010/11, Uganda 2010/11.

Fig. 1 graphically explores within-country inorganic fertilizer and agro-chemical use statistics across the six LSMS-ISA countries. It remains to be established whether such variation corresponds with differences in the profitability of input use or any other important driver. Analysis of the marginal costs and benefits of using modern inputs (e.g., Sheahan et al., 2013 on Kenya and Liverpool-Tasie et al., 2015 on Nigeria) – infeasible in the descriptive, cross-sectional work undertaken here – would help determine whether this variation corresponds with efficient allocation of inputs according to variation in prices and productivity impacts or if various constraints better explain the considerable heterogeneity we observe among and within regions intra-nationally.

### 3.4 There is surprisingly low correlation between the use of commonly “paired” modern inputs at the household- and, especially, the plot-level

It is commonly thought that modern inputs are seldom adopted in isolation since the complementarity between particular sets of inputs makes adopting them together advantageous for farmers, as well as the fact that inputs are generally sold alongside each other at input shops or provided together via government subsidy programs. If there are agronomic (or other) synergies among modern inputs, it is believed, then farmers will use them together, especially if farmers behave “efficiently.” For example, some modern seed varieties are bred to respond better when paired with inorganic fertilizer (Ellis, 1992, Nyangena and Juma, 2014). The entire integrated soil fertility management (ISFM) paradigm is built around the belief that inorganic and organic fertilizer should be used together to improve both the nutrient availability and absorption capacity of the soil (Place et al., 2003, Vanlauwe et al., 2010, Vanlauwe et al., 2011). Furthermore, the use of inorganic fertilizer may increase the presence of more weeds on the plot, necessitating the combined use of herbicide. Irrigation systems help to secure necessary soil moisture for efficient inorganic fertilizer use and improved seed varietal growth (Yilma and Berger, 2006). Rosegrant et al. (2014), using a crop model incorporating climate change scenarios, predict massive gains to combining nitrogen at efficient levels with irrigated maize and rice in SSA.

Using simple correlation coefficients of binary input use decisions at the household level, we find a surprising dearth of synergistic use of modern inputs. Conditional on using inorganic fertilizer, there is low correlation with use of other inputs in most cases. For example, inorganic fertilizer using households are highly likely also to use an organic fertilizer only in Ethiopia and Niger, suggesting that households in most other countries view the two as substitutes instead of complements, underscoring the ongoing challenge of promoting ISFM. Users of improved seed varieties are very likely also to use inorganic fertilizer in Ethiopia, but not in Niger, the only two countries where the data allow us a full look at improved seed varietal use across all crop types.^7^ Agro-chemicals and inorganic fertilizers are often used together at the household level (except in Uganda and Niger), implying a relatively high amount of chemicals used in agriculture for these households. Owning agricultural equipment and having some fields under irrigation also are not consistently highly correlated across countries, and irrigation and machine ownership, separately, are only highly correlated with inorganic fertilizer use in half of the countries. Ethiopia seems to have the highest amount of joint correlation, and Uganda the least. Generally speaking, we find some areas of low correlation between “paired” input use patterns, suggesting that there are still yield gains to be exploited by using inputs together on farm.^8^

Going further, because the hypothesized complementarities among inputs are primarily biophysical, we would expect that households use synergistic inputs together on the same plot, not just on the same farm. At the plot level, however, we find far less correlation than at the household level, with only a handful of instances where two inputs are used together in high percentages and with no noticeable patterns across countries. There are a few instances where chemicals – inorganic fertilizers and agro-chemicals like pesticides – are used together, providing further evidence that their use may be higher than policy makers and analysts recognize.

**Fig. 2:**
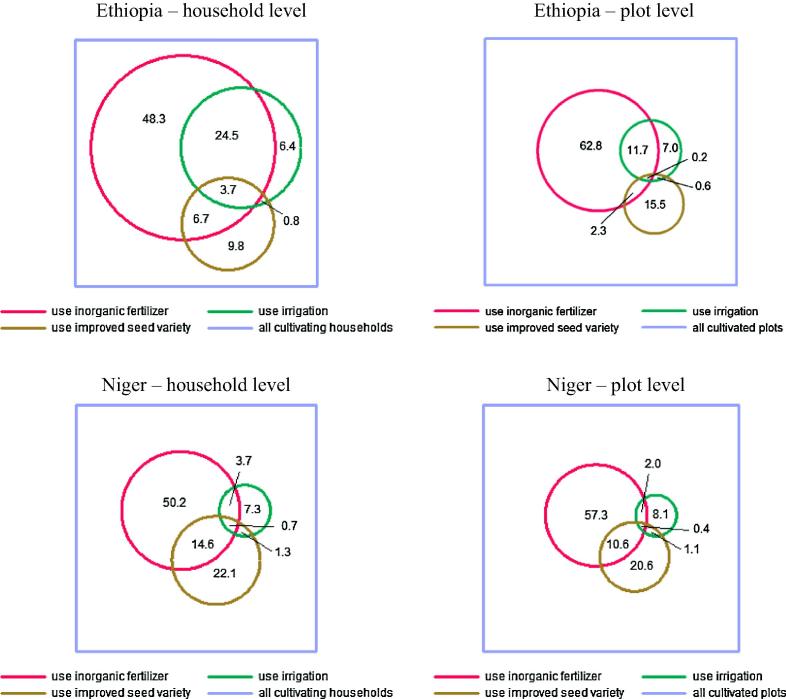
Venn diagrams of three-way input use in Ethiopia and Niger.

*Source:* Authors’ calculations using the Living Standards Measurement Study Integrated Surveys on Agriculture: Ethiopia 2011/12, Malawi 2010/11, Niger 2011/12, Nigeria 2010/11, Tanzania 2010/11, Uganda 2010/11. Notes: These figures were created using the “pvenn” user-written command in Stata. The areas of the circles proportionally represent population size relative to the full sample of cultivating households. The percentages in are conditional on using any one of the three included inputs (i.e., exclude the population that does not use any of the three inputs) and are not weighted.

In order to depict graphically the decreasing level of correlation when moving from the household to plot level, Fig. 2 displays Venn diagrams reporting the full set of conditional probabilities for use of inorganic fertilizer, improved seed varieties, and irrigation—representing an interesting mix of short and potentially longer term investments and may provide the largest gains when paired—at the household and plot level in Ethiopia and Niger. The overlapping area, representative of the use of at least two of the three inputs, is relatively small at the household and plot level. When burrowing down to the intersection of all three inputs, less than 4% of households use all three inputs in Ethiopia and less than 1% uses them together in Niger, conditional on using at least one of the three. And less than 1% of plots in either country receive all three inputs, again conditional on using one input. This implies that the small minority of households that are using multiple modern inputs tend to spread them across plots rather than concentrating them on single plots. This behavior has gone largely unstudied to date and raises important questions about prospective untapped productivity gains from coordinated modern inputs use, with implications for extension programs and policies aimed at promoting efficient input uptake and use.

### 3.5 Input intensification is happening for maize in particular

A major strong point of the LSMS-ISA data collection effort is the assembly of detailed plot-level information, including all of the crops and their relative share of plot area. While our categorization is rough, we attempt to isolate the “most important” crop on the plot, defined as comprising at least 50% of the plot area under cultivation. These categorizations offer an admittedly-imperfect attempt to categorize plots by the types of crops grown on them, but are arguably no more arbitrary than other classification schemes used in the literature.

Apart from the fact that the “other” category contains the largest number of plots in most countries, pointing to the high degree of mixed and intercropping in SSA, another striking pattern is that plots with mostly maize are among those most likely to receive a modern input and with the highest application amounts. The two cases where maize plots are not always the most intensively cultivated – although still among the highest – are Ethiopia and Malawi where teff (as also reported in Minten et al., 2013) and tobacco plots, respectively, receive more inputs. Contrary to much prevailing prior belief, agro-chemicals do not appear confined to plots with horticultural or cash crops (which would fall into the “other” category), with relatively high percentages also applied on plots containing mostly grains. This finding mirrors Williamson et al. (2008), who observed a very high rate of pesticide use not just on cash crops and vegetables, but also on staple crops. In plot-level regression analysis using data from all countries (with household level fixed effects applied), we find that pure stand and intercropped maize fields are significantly more likely to receive inorganic fertilizer than non-maize-dominated plots.^9^

Moreover, plots that include (but are not necessarily dominated by) a major cash crop – less than one quarter of the total observed – are generally no more likely to receive modern agricultural inputs of any sort. We selected a set of cash crops from the full list of available crops in the survey.^10^ After separating plots this way, we find no evidence that plots with cash crops are more likely to receive one of these modern inputs. In fact, plots *without* cash crops are more likely to receive inorganic fertilizer in Ethiopia, Malawi, and Nigeria.^11^ This finding further elevates the previous result that plots with staple grains, maize in particular, are actually receiving the lion’s share of the input use, possibly due to the focusing of extension on food crops.

The attention to maize intensification also extends to findings on use of improved and commercial seed. As shown in Table 6, 25–40% of maize cultivating households purchased new maize seed in the last main agricultural season. Of the few places where we observe full improved seed variety statistics, nearly one-quarter of maize cultivating households in Ethiopia and over half in Malawi used an improved variety in the main growing season. These findings suggest more widespread participation of African agricultural households in modern input distribution systems than has been widely recognized. The weight of the evidence suggests that maize may be “on the move” in Africa, an especially important finding given the significance of maize as a food security crop for many households in the region. Niger, however, is largely removed from this discussion given the very small contribution of maize to household production and consumption.

### 3.6 An inverse relationship consistently exists between farm or plot size and input use intensity

**Fig. 3:**
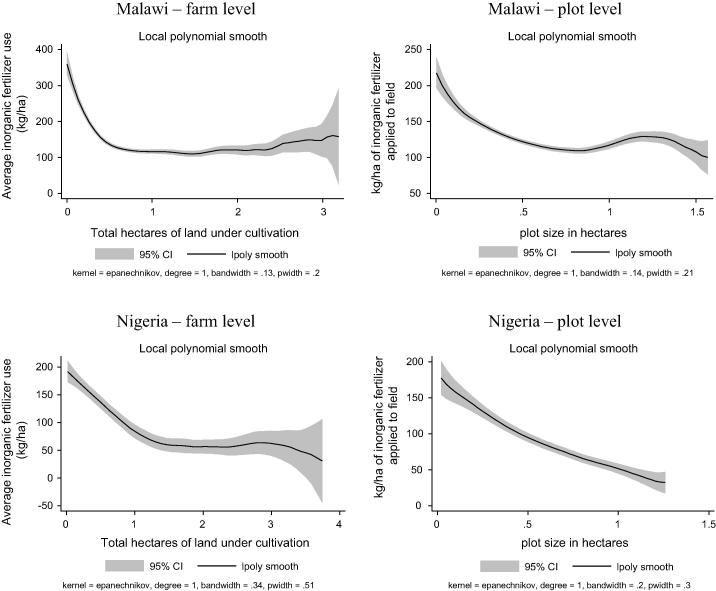
Local linear non-parametric regression of average total fertilizer use per hectare by total number of hectares cultivated by household in main season.

*Source:* Authors’ calculations using the Living Standards Measurement Study Integrated Surveys on Agriculture: Ethiopia 2011/12, Malawi 2010/11, Niger 2011/12, Nigeria 2010/11, Tanzania 2010/11, Uganda 2010/11. Notes: Farm size is defined as the total number of hectares under cultivation in the main season and does not include rented out or fallow land. Multiply imputed plot size variables, following the discussion in Section 2, are used in the aggregation to the household level. Farm and plot sizes observations above the 99th percentile in each country are excluded from these figures. Inorganic fertilizer application rates are representative of total inorganic fertilizer, not nutrients. A full set of graphs for all countries can be found in Sheahan and Barrett (2014).

The inverse (negative) relationship between farm size and productivity is a well-studied and fairly entrenched phenomenon in the agricultural development literature (e.g., Carter, 1984, Feder, 1985, Barrett et al., 2010). What is less well-documented with data from farmers’ fields is the relationship between input use intensity and farm size (important exceptions include Croppenstedt et al., 2003, Doss, 2003). We corroborate that latter pattern using the LSMS-ISA data and find that this relationship is robust even when controlling for farm-level effects and possible measurement error that can be corrected using GPS-verified plot sizes. Using non-parametric local polynomial regressions, Fig. 3 shows examples from two countries – Malawi and Nigeria – both of which well-demonstrate the consistent negative relationship between farm size, defined as total area under cultivation in the most recent main season, and household-averaged inorganic fertilizer application rates. Some of the most negative relationships occur in the countries that have larger average farm sizes, like Niger and Tanzania. On the other hand, in Malawi, where average farm sizes are the smallest, the relationship holds only for the 90% of households with farm sizes less than 1.5 ha. On the contrary, in Ethiopia farm sizes need to approach three hectares (around the 95th percentile of the farm size distribution) before a statistically significant negative relationship sets in, making it the outlier in the group. Moreover, in Ethiopia and Uganda, there is a range over which the relationship is (mildly) positive before falling again.

Less commonly investigated is this same relationship at the plot level. When performing the same nonparametric regressions at that level of disaggregation instead, the inverse relationship is, perhaps surprisingly, even stronger than at household level in virtually all cases (Fig. 3), with Ethiopia as the notable exception, as above. The powerful implication is that inter-household variation in the shadow price of inputs and outputs based on endowments, distance to market, etc. cannot explain the inverse size-input use relationship, as much of the existing literature implies when suggesting that both equity and efficiency goals might be advanced by progressive land transfer programs that would redistribute land from larger land owners to those with smaller holdings. Consistent with the findings of Barrett et al. (2010), the striking within-household inverse relationship raises novel puzzles about farmers’ behaviors and the input productivity implications that have yet to draw much research attention.

### 3.7 Farmers do not significantly vary input application rates according to perceived soil quality

One would expect that farm management practices would follow from the knowledge a farmer has about the environment in which they operate. One important characteristic of the operating environment that should affect input use decisions is soil quality since, for example, it is well known that the responsiveness of crops to fertilizer application depends on the quality and fertility of the soil (e.g., Zingore et al., 2010, Zingore, 2011). Even within a given farm, evidence suggests that productivity can differ immensely between plots (Tittonell et al., 2005), so too, then we would expect soil fertility status also to vary. Moreover, household perceptions of soil quality may influence fertilizer application rates (Marenya and Barrett, 2009) and be influenced, in turn, by previously observed crop yields (Marenya et al., 2008). We test these claims in the three countries (Malawi, Tanzania, Uganda) where the LSMS-ISA surveys elicited farmer perceptions of soil quality by plot.

Unexpectedly, modern input use and rates—particularly for inorganic fertilizer, agro-chemicals, and irrigation—are virtually identical or only slightly different among plots categorized by survey respondents as good, average, and poor quality.^12^ Using simple descriptive statistics, farmers do not appear to adjust input application rates to accommodate their perceptions of plot quality. Regression analysis of within-farm variation on more than 26,000 plots on 14,000 farms holding constant observable and unobservable farm-level factors reveals that plots deemed “average” or “poor” in quality are statistically significantly more likely to receive inorganic fertilizer applications than are plots categorized as “good,” however these variables explain only a tiny amount of within-household fertilizer allocation decisions.^13^

Similar to soil quality more generally, erosion is seen as one of the avenues through which soils degrade and lose their inherent productivity levels. Erosion is also the consequence of soil fertility depletion and, therefore, can act as another proxy for poor soil quality. Erosion control (e.g., through contour ridges, rock lines, vegetative bands, living hedges), then, is seen as a vehicle for maintaining soil fertility, particularly when paired with fertilizer use and legume intercropping (Morris et al., 2007).

Analogous to the respondent-perceived soil quality story, farmers do not appear to make tremendously different input use decisions across eroded and non-eroded plots in the four countries (Niger, Uganda, Malawi, Tanzania) in which respondent perceived erosion-status was elicited. Only in Niger and Uganda, the two countries with the lowest inorganic fertilizer use rates, do we observe higher unconditional fertilizer application rates for non-eroded plots. Organic fertilizer decisions do not appear to be made based on the erosion status of a plot. This suggests that farmers view organic fertilizer application neither as an investment in improving soil health nor as a waste of scarce resources. Interestingly, outside of Malawi, where the differences are practically insignificant, eroded plots are slightly more likely to be irrigated than are non-eroded ones.

To the extent that crop response rates to particular inputs will vary significantly by soil quality and erosion status, these findings may signal a knowledge gap among farmers and raise important questions about the accuracy and drivers of farmer perceptions of soil quality. The use of organic inputs in particular may also increase the quality of the soil and productivity of the land over time, so the fact that “poor” plots are no more likely to receive organic fertilizer in Uganda and eroded plots are not statistically significantly more likely to receive organic fertilizer application in any of the four listed countries seems cause for concern. Together, these findings suggest a need for renewed efforts at extension programming around soil fertility (beyond just soil type) and input use and, possibly, the need to invest in inexpensive but accurate soil quality tests.

### 3.8 Few households use credit to purchase modern inputs

Because cash resources may be limited for smallholder farmers or cash inflows do not arrive when inputs need to be purchased, access to credit can be an important catalyst to input use and subsequent agricultural productivity gains. For example, Matsumoto and Yamano (2011) find that having access to fertilizer credit increases teff yields by 37% in Ethiopia. Because of poorly developed financial markets and the high risks associated with providing credit to smallholder farmers, credit is widely thought to be used only minimally throughout SSA and, therefore, to act as a major constraint to input use (e.g., Croppenstedt et al., 2003, Zerfu and Larson, 2010).

In all LSMS-ISA countries except Ethiopia, less than 1% of cultivating households used credit—either formal or informal—to purchase improved seed varieties, inorganic fertilizer, or agro-chemicals. In Ethiopia, where there exist widespread input credit guarantee schemes operated by cooperatives (Matsumoto and Yamano, 2011), nearly 25% of cultivating households claimed to receive some type of “credit service,” although we cannot be sure whether this is for agriculture or other household purchases. Apart from this generic question, we observe about 5% of households acquiring credit to pay for maize seed in particular, but do not observe similar statistics for other inputs. In Malawi, Niger, and Uganda, we observe credit use specifically for inorganic fertilizer and agro-chemicals. But for all three countries and both inputs, less than 1% of cultivating households claim to have received credit for the purchase of either of these inputs. In Nigeria, since we do not specifically observe improved seed purchases, we lump together all credit obtained for purchasing any seed type. Even then, the percent of farmers using credit to purchase seeds is less than 1%, just like inorganic fertilizer. In Tanzania, we also observe seed credit usage and find, again, less than 1% participation.

The cross-country, nationally representative data reinforce widespread perceptions of the weakness of agricultural input credit markets in the region. While saying nothing of the relative need for credit to purchase the included inputs, our findings show that much scope remains for deepening rural financial markets, despite recent advances in money transfer systems based on mobile phone platforms, the proliferation of microfinance institutions, etc. Indeed, considerable room for research exists in identifying and examining cases of successful agricultural input credit schemes.

### 3.9 Gender differences in input use exist at the farm and plot level

The headship of the household is one characteristic often believed to limit modern input use. A number of studies find that lower levels of productivity and income among female-headed households can be partially attributed to lower access to improved inputs (e.g., Djurfeldt et al., 2013, FAO, 2011), although a range of country and within-country specific factors remain similarly instrumental in perpetuating this gap (Kilic et al., 2015, as well as the entire special issue of *Agricultural Economics* in May 2015). In the LSMS-ISA countries we find that male-headed households are indeed statistically significantly more likely to use modern inputs across almost all countries and input types. That result holds in both simple descriptive statistics and multivariate regression analysis holding some other important covariates constant.

As an extension of the relationship between the gender of the household head and input use, we also examine the patterns among plots managed by different members of respondent households, another often over-looked and potentially illuminating area of within-farm input use distribution. In the LSMS-ISA surveys, about 18% of plots are owned or managed by females across all countries, ranging from 8% in Nigeria to 33% in Uganda.^14^ In most countries, plots managed or owned by men, the vast majority of all plots, are statistically significantly more likely to receive inorganic fertilizer and in higher amounts. Men tend to use more agro-chemicals in certain countries (Malawi, Nigeria, Tanzania) and irrigation in others (Ethiopia, Niger, Nigeria). When pooling across countries and performing simple regression analysis to predict binary inorganic fertilizer use, controlling for (among other things) our plot-type classification based on included crops, we find that male-managed plots remain statistically significantly more likely to receive inorganic fertilizer. Because most plots managed or owned by females are also found in female-headed households, we perform robustness checks on these results by limiting our sample to only male-headed households. Indeed the statistical significance of these relationships remains unchanged in all countries except Malawi (apart from the case of agro-chemicals). Overall, however, the gender of the plot manager or owner does not appear to be a major determinant of input use in Ethiopia, Tanzania, or Uganda unlike in Malawi, Niger, and Nigeria.

Our findings mimic the related descriptive work on the “gender gap” in African agriculture using the LSMS-ISA data by the World Bank and ONE Campaign (2014), revealing that even when the gap in access to modern inputs is closed, the returns to the use of those inputs is less for women than men, pointing to cultural norms, market failures, and institutional constraints that presumably suppress the productivity gains for women. These prospective mechanisms, and gender differences in modern agricultural input use and associated yield increases more broadly, both among and within households, merit more attention as they may lead to needless productivity losses and food insecurity. The related discussion of labor input distribution by gender across agricultural tasks using these same data sets is included in this issue (Palacios-Lopez et al., 2015).

### 3.10 National-level factors explain nearly half of the farm-level variation in inorganic fertilizer and agro-chemical use

A huge literature exists that promotes one set of variables as the most important reason for the “adoption” or use of a particular input, be it biophysical, infrastructure, market, socio-economic, or otherwise. Having so many observations across multiple countries with similar covariates allows us the unique opportunity to test which of these variables or classes of variables is most strongly associated with variation in input utilization. Because our analysis only includes one cross-section of observations in each country, the relationships we uncover using regression techniques are still mere correlations and do not have a causal interpretation, especially because there remains considerable unobserved heterogeneity for which we cannot account. Using standard ordinary least squares (OLS) regression, we estimate separate linear probability models for inorganic fertilizer and agro-chemical use at the household level, pooling observations across all six LSMS-ISA countries. We then calculate Shapley values, which decompose the explained variance (measured by R^2^) of those regressions into contributions over particular groups of regressors (Huettner and Sunder, 2012), in other words the mean marginal contribution of each variable or group of variables to the overall regression model R^2^.

Table 7 reports our regression estimates and estimated Shapley values for the binary inorganic fertilizer use decision. Quite surprisingly, the overwhelming amount of variation, indeed nearly half (45%), is accounted for by the country dummy variables.^15^ Even controlling for a wide range of important observable household-level and agro-ecological variables, some combination of other policy, institutional, or macroeconomic variables explain most of the micro-scale variation in inorganic fertilizer use in this unprecedentedly large sample of over 22,000 households. Since our dependent variable is the binary input use decision, differences in survey design, which may lead to differences in measurement of continuous input volumes, cannot plausibly account for the importance of the country-level variables. This is a significant finding, as clearly the policy and operating environments facilitated by governments matter. This underscores the importance of processes such as the Comprehensive Africa Agriculture Development Programme initiated by the New Partnership for Africa’s Development.

Biophysical variables account for 24% of the explained variation in fertilizer use, followed by farm characteristics that together account for 16%, market and accessibility variables account for nearly 10%, and household socio-economic variables less than 4%.^16^ It is perhaps surprising, given other findings on gender-based differences in particular, that household socioeconomic status actually explains little of the observed inter-household variation in modern input use rates, far less than national-scale, biophysical and market-related variables. The fact that geography (explained by the country level dummy variables) and other biophysical characteristics (accounting for a combined 70% of variation) matter so much to the fertilizer use decisions mirrors, to a large extent, findings by McCord and Sachs (2013) on the importance of the same factors in explaining variations in macroeconomic development conditions across countries. This is an especially striking finding that signals the critical importance of catering the policy and institutional environment to biophysical realities for ushering in a Green Revolution in SSA.

## 4 Conclusions

In this paper we undertake a descriptive cross-country comparison of the six Living Standards Measurement Study Integrated Surveys on Agriculture (LSMS-ISA) countries in SSA (Ethiopia, Malawi, Niger, Nigeria, Tanzania, and Uganda) to revisit the current agricultural input landscape. At a time when governments and donors are redoubling efforts to stimulate a Green Revolution in Africa, it is imperative to interrogate longstanding conventional wisdoms, especially in light of substantial changes in both governments’ policies and in the overall contexts within which farmers make input use decisions. Sweeping general statements, often based on outdated or statistically non-representative data, like “modern input use in Africa is low,” do little to advance constructive policy analysis or debate. The descriptive evidence we summarize from newly available, large-scale, nationally-representative, and cross-nationally comparable data underscore the need for basing policy and business decisions on more nuanced and up-to-date assessments and, indeed, continuing to invest in good quality national agricultural statistics.

We learn, for example, that some longstanding beliefs remain largely true. Irrigation use and mechanization levels remain low in SSA agriculture. Women farmers use far fewer inputs than men. The use of credit to purchase agricultural inputs is nearly non-existent. And a strong inverse relationship exists between farm, or even plot, size and input use intensity. But other widespread beliefs about agricultural input use in SSA appear in need of updating and further exploration. For example, while the use of inorganic fertilizer and agro-chemicals remains relatively low on average, use rates are actually quite high in some countries and regions within countries. This may relate to the fact that input use is no higher on cash crop plots than on those cultivated mainly with staple cereals, particularly maize, a staple crop in most of the survey countries. Concerted efforts to stimulate modern input use, especially around maize, seem to be experiencing some success that has perhaps gone under-recognized.

Yet, even with perhaps-higher-than-previously-recognized rates of use of modern agricultural inputs, these inputs are rarely used together on plots, despite widespread evidence of agronomic synergies from, for example, coupling irrigation, improved seeds, and inorganic fertilizer use. Similarly, despite considerable agronomic evidence of variable returns to input use on soils of different quality, there is negligible variation in input use by farmer self-reported soil quality, or even by plot-level erosion status. These findings suggest significant, and somewhat puzzling, foregone productivity gains that merit deeper exploration.

While this paper provides us with, at a minimum, nationally-representative descriptive statistics derived from micro-data that have been largely absent from input intensification debate, it cannot speak to critically important issues of the causal mechanisms behind the patterns we describe, nor to dynamics of diffusion and disadoption of agricultural inputs, much less to implications for profitability, welfare gains, bargaining power, etc. that are most directly relevant to policy. And policy matters a lot. Indeed, we find that country-level factors, like policy differences, explain far more of the predictable variation in agricultural input use decisions than do biophysical, market, farm, or household socioeconomic characteristics. Since our findings can only go so far in uncovering key inter-country differences and certainly cannot identify the specific policies that have caused expanded input use in particular areas, mainly our results open up a range of important new policy research questions amenable to exploration with the publicly-available LSMS-ISA panel data, especially when merged with geo-referenced secondary data series.

The challenge of sparking a Green Revolution in Africa requires a solid foundation in descriptive evidence on contemporary African farmers. Our findings may be best conceptualized alongside related work on the correlates with intensification patterns in the region (Binswanger-Mkhize and Savastano, 2014), labor productivity differentials (McCullough, 2015) as potentially driven by input use, and the existence of multiple market failures (Dillon and Barrett, 2014). We hope this paper, coupled with these related pieces, contributes in some measure to provide a fruitful base for constructive policy discussions and investments.

## Figures and Tables

**Table 1:** Number of households and plots included in analysis versus overall survey sample.

Country	Survey year	Name of main season	Overall LSMS-ISA survey sample		Sub-sample used in this analysis (main season)			
			No. of hh	% of hh in “rural” areas	No. of hh	% of overall survey sample in analysis	% of hh in “rural” areas	No. of plots
Ethiopia	2011/12	Meher	3969	98.9	2852	86.6	99.7	23,051
Malawi	2010/11	Rainy	12,271	84.4	10,086	83.2	93.5	18,598
Niger	2011/12	Rainy	3968	61.2	2208	77.9	93.8	6109
Nigeria	2010/11	–	5000	59.0	2939	49.9	84.6	5546
Tanzania	2010/11	Long rainy	3924	69.1	2372	66.6	85.9	4794
Uganda	2010/11	First	2716	83.5	2108	73.8	93.7	4289

Sample size across countries			31,848	76.0	22,565	73.0	91.9	62,387

Notes: All surveys are nationally representative except Ethiopia, which was only conducted in rural areas (with a few households in “small towns”). In Ethiopia, only one of the two seasons is captured in the surveys. “Rural” areas are defined differently across countries. The sample sizes described above are not weighted, but percentages are. The aggregated sample size across the six countries includes simple summations and unweighted averages.

**Table 2:** Inorganic fertilizer use statistics from macro and LSMS-ISA data.

Country	FAOSTAT^a^				LSMS-ISA^b^							
	Use (kg/ha)				% of cultivating households using	Use (kg/ha) across all households		Use (kg/ha) across only fertilizer using households				
	Mean N	Mean P	Mean K	Mean nutrients		Mean total	Mean nutrients	Mean total	Mean N	Mean P	Mean K	Mean nutrients
Ethiopia	10.4	10.8	0.0	21.2	55.5	45.0	25.2	81.0	23.0	22.5	–	45.5
Malawi	23.1	4.5	4.3	31.9	77.3	146.0	56.3	188.8	53.1	19.4	0.4	72.8
Niger	0.3	0.1	0.1	0.5	17.0	4.5	1.7	26.3	7.6	2.6	–	10.3
Nigeria	2.0	0.3	0.3	2.6	41.4	128.2	64.3	310.1	93.9	30.8	30.8	155.5
Tanzania	4.4	0.6	0.7	5.7	16.9	16.2	7.7	95.6	32.0	7.0	6.6	45.6
Uganda	0.7	0.3	0.3	1.3	3.2	1.2	0.7	37.5	11.5	8.3	1.0	20.7

Average	6.8	2.8	1.0	10.5	35.2	56.9	26.0	123.2	36.9	15.1	9.7	58.4

Notes: Nutrient values represent the actual nutrient content in all applied fertilizers. The “average” row includes simple (unweighted) averages across the statistics reported at the country level.

a FAOSTAT data from 2010. Cultivated land defined as arable land plus land under permanent crop. See more details at FAOSTAT.

b Authors’ calculations using the Living Standards Measurement Study Integrated Surveys on Agriculture: Ethiopia 2011/12, Malawi 2010/11, Niger 2011/12, Nigeria 2010/11, Tanzania 2010/11, Uganda 2010/11.

**Table 3:** Agro-chemical use statistics from macro and LSMS-ISA data.

Country	Literature review^a^		LSMS-ISA^b^			
	% hh using	Source	% of cultivating hh using	By type		
				Pesticide	Herbicide	Fungicide
Ethiopia	21	Taffesse (2008)	30.5	8.4	27.2	3.5
Malawi	3	Zezza et al. (2007)	3.0	–	–	–
Niger	–	–	7.8	1.9	0.7	5.5
Nigeria	10.5	Akramov (2009)	33.0	18.2	21.9	–
Tanzania	–	–	12.5	–	–	–
Uganda	3	Okoboi (2010)	10.7	–	–	–

Average			16.3	–	–	–

Note: FAOSTAT data on agro-chemical use includes application rates, which are not as reliably generated using the LSMS-ISA data. For that reason, we exclude those statistics from this table. The “average” row includes simple (unweighted) averages across the statistics reported at the country level. The breakdown by type of agro-chemical in the LSMS-ISA statistics is only included for those countries with full statistics for pesticides, herbicides, or fungicides.

a Review of literature.

b Authors’ calculations using the Living Standards Measurement Study Integrated Surveys on Agriculture: Ethiopia 2011/12, Malawi 2010/11, Niger 2011/12, Nigeria 2010/11, Tanzania 2010/11, Uganda 2010/11.

**Table 4:** Irrigation incidence statistics from macro and LSMS-ISA data.

Country	AQUASTAT/FAO^a^			LSMS-ISA^b^		
	Year	Total irrigated land (ha)	% of land	Total cultivated land under irrigation by smallholders (ha)	% of all cultivated land under irrigation by smallholders	% of households with at least some irrigation on farm
Ethiopia	–	–	–	163,087	1.3	8.7
Malawi	2006	26,900	0.79	4090	0.2	0.4
Niger	2005	65,610	0.46	136,383	1.4	6.9
Nigeria	2004	218,800	0.61	274,681	2.5	4.1
Tanzania	–	–	–	239,493	1.8	3.6
Uganda	2010	12,450	0.14	174,972	3.5	3.9

Average				165,451	1.8	4.6

Note: All irrigation values related to the LSMS-ISA data are drawn from the main season only. The “average” row includes simple (unweighted) averages across the statistics reported at the country level.

a AQUASTAT/FAO (various years) area actually irrigated country data sheets for most recent year with available data. Percent of land values calculated using total arable land plus permanent crop land from FAOSTAT. See more details at FAOSTAT. For more on the AQUASTAT data and project, see Frenken (2005).

b Authors’ calculations using the Living Standards Measurement Study Integrated Surveys on Agriculture: Ethiopia 2011/12, Malawi 2010/11, Niger 2011/12, Nigeria 2010/11, Tanzania 2010/11, Uganda 2010/11.

**Table 5:** Mechanization level statistics from macro and LSMS-ISA data.

Country	FAOSTAT^a^		LSMS-ISA^b^				
	Year	Number of tractors in country	Number of tractors in country	% of hh that own a tractor	% of hh that rent a tractor	% of hh that own any equip	% of hh that rent any equip
Ethiopia	–	–	–	–	–	73.6	–
Malawi	1968	692	707	<0.1	<0.1	0.8	1.1
Niger	2006	375	6286	0.3	0.2	77.5	13.6
Nigeria	2007	24,800	449,688	1.6	–	9.4	–
Tanzania	2002	21,207	170,250	2.2	3.0	16.4	19.1
Uganda	1977	2076	11,574	0.2	0.5	13.6	15.1

Average			127,701	1.1	1.2	31.9	12.2

Notes: For the number of tractors summation, the full sample – not what is found in Table 1 – is used in order to more accurately predict the number of tractors at the national level. The “average” row includes simple (unweighted) averages across the statistics reported at the country level.

a FAOSTAT. For total and rural population definitions, see FAOSTAT.

b Authors’ calculations using the Living Standards Measurement Study Integrated Surveys on Agriculture: Ethiopia 2011/12, Malawi 2010/11, Niger 2011/12, Nigeria 2010/11, Tanzania 2010/11, Uganda 2010/11.

**Table 6:** Commercially purchased and improved maize seed statistics from macro and LSMS-ISA data.

Country	DIIVA^a^		LSMS-ISA^b^		
	% of land under improved maize seed varieties	Number of households that cultivate maize	Improved maize seed varieties		Commercially purchased maize seeds
			% of cultivating households using improved variety	% of area cultivated with maize under improved variety	% of cultivating households that purchased commercial maize seed (irrespective of variety)
Ethiopia	27.9	1760	23.7	33.7	40.7
Malawi	43.0	9861	56.2	40.5	31.5
Nigeria	95.0	1247	–	–	24.0
Tanzania	35.4	1715	–	–	29.8
Uganda	54.0	1246	–	–	36.6

Notes: Commercial seed can be of any variety. Niger is excluded due to the unimportance of maize and the inconsistency in English translation of survey instrument and how the data was supposedly collected from respondents. Similar statistics for other crops can be found in Sheahan and Barrett (2014).

a CGIAR’s Diffusion and Impact of Improved Varieties in Africa (DIIVA) project 2009 values.

b Authors’ calculations using the Living Standards Measurement Study Integrated Surveys on Agriculture: Ethiopia 2011/12, Malawi 2010/11, Niger 2011/12, Nigeria 2010/11, Tanzania 2010/11, Uganda 2010/11.

**Table 7:** Decomposition of binary inorganic fertilizer use decision at household level.

		Coeff. Est.	Sig	Std. Err.	Shapley
***Bio***-***physical variables***					
+ Annual precipitation (mm)		0.0000356	⁎⁎⁎	0.000012	0.99
+ Elevation (m)		0.000346	⁎⁎⁎	0.00000962	7.98
+ Nutrient availability of soil		(categorical)	–		2.40
+ Maximum greenness (EVI) in growing season		−0.05217		0.0562836	1.23
+ Agro-ecological zones		(categorical)	–		11.30

***Socio***-***economic variables***					
Consumption (per AE) quintiles		(categorical)	–		2.55
Sex of hh head (1 = female)		−0.02466	⁎⁎⁎	0.0067751	0.27
Household size		0.012796	⁎⁎⁎	0.0011004	0.63
Household dependency ratio		−0.00387		0.0033838	0.18

***Farm operation characteristic variables***					
Size of hh land under cultivation (ha)		−0.00052		0.0013721	1.02
Number of crops produced by hh		0.024381	⁎⁎⁎	0.0017476	1.49
Cash crop produced by hh (1 = yes)		0.043524	⁎⁎⁎	0.0065025	2.09
Maize produced by hh (1 = yes)		−0.10684	⁎⁎⁎	0.0079161	11.85

***Market and accessibility variables***					
+ Distance to nearest market (km)		−0.00044	⁎⁎⁎	0.0000889	8.23
+ Distance to nearest major road (km)		−0.00048	⁎⁎	0.0001854	1.06
Fertilizer price per kg (in USD)		0.000092		0.0003704	0.45
Main grain price per kg (in USD)		0.50949	⁎⁎⁎	0.0736567	0.89

***Country dummy variables***		(categorical)	–		45.40

Notes: n = 22,214 households; overall R^2^ = 0.393. Variables with a plus sign (+) are merged from a number of geo-referenced data sets mentioned in Section 1. Certain geo-referenced and aggregate variables are not currently available for Uganda 2010/11, so the same values for the 2009/10 round are used in their place. The main grain price is maize in all countries except Niger where the price of millet is used in its place. To standardize prices of fertilizer and grain, we use official exchange rates (to USD) from the World Bank. Household level weights are not used (meaning households from Malawi are over-weighted in these results). This table was created using the “rego” user-written command in Stata.

^*^ p < 0.1.

⁎⁎⁎ p < 0.01.

⁎⁎ p < 0.05.

## Footnotes

1 The surveys are also population-representative at various sub-national levels, differing by country. We refer interested readers to the individual survey documentation (all available on the LSMS-ISA website) for more details.

2 While we use the term “plot” throughout this analysis for simplicity, the actual unit of land described in each of these surveys may differ: Ethiopia-field within parcel by holder; Malawi-plot; Niger-parcel within field; Nigeria-plot; Tanzania-plot; Uganda-parcel (aggregating input use across plots on a parcel).

3 For much more detail on our data cleaning protocols, see Appendix 2 in Sheahan and Barrett (2014).

4 We make no attempt to separate total input use from commercially purchased and subsidized input use given the inconsistency with which data on input sources are collected and recorded across countries. We note, however, that only 5% of cultivating households in Nigeria claimed to receive fertilizer from the government or a politician while around 53% of households in Malawi and 9% in Tanzania claimed to receive a voucher for fertilizer in the main growing season.

5 There are many possible reasons for the mismatches in the remaining two countries: differences in land area definition used between the two estimates (cultivated land versus land available for cultivation, including temporary fallow); differences in time frame used (calendar year versus main agricultural season); differences in included sample (smallholders versus all of agricultural production); etc. This paper does not concern itself with reconciling these differences in methodology.

6 Note that most of the irrigation in Niger is concentrated in the *contre* season, not the main season, so the statistics included here underestimate the total incidence of irrigation used year round.

7 Because the “improved seed variety” category lumps all seeds together in Ethiopia and Niger, we separately explored the correlation between improved maize seed usage in Ethiopia and Malawi (maize production is very minor in Niger). We find that, of the plots with improved maize seeds planted on them, 82% of plots in Malawi and 88 in Ethiopia have inorganic fertilizer also applied; those households are truly exploiting the gains from joint use of improved maize seed and inorganic fertilizer.

8 Moreover, receiving a government subsidy for one input does not automatically ensure that the use of another will follow. In Malawi, for example, around 60% of inorganic fertilizer-using households used an improved maize variety, virtually the same value across recipients and non-recipients of fertilizer subsidies.

9 Note, however, that households that do not produce maize are statistically significantly more likely to use inorganic fertilizer anywhere on their farm (see Table 7), illuminating the importance of within-farm analysis.

10 Cash crops are defined differently in each country, with the distinction made by the LSMS-ISA team at the World Bank. The cash crops included here are: Ethiopia-coffee, cotton, groundnuts; Malawi-tobacco, groundnuts; Niger-cotton, groundnuts; Nigeria-cocoa, groundnuts, cotton, palm oil; Tanzania-coffee, cotton, cashew nuts, tobacco, coconut, groundnuts; Uganda- coffee, cotton, groundnuts.

11 At the same time, households that produce at least one of these cash crops are statistically significantly more likely to use inorganic fertilizer in some amount on their farm (see Table 7).

12 One, perhaps unsurprising, side finding is the relatively low number of plots currently under cultivation that are characterized as “poor” or “bad” by the respondents. This implies that households are only cultivating plots that they consider of higher quality.

13 For detailed regression results, see Sheahan and Barrett (2014).

14 Plot-level operators take different names across surveys. We use the following: Ethiopia-holder; Malawi-manager; Niger-owner; Nigeria-manger; Tanzania-manager; Uganda-manager.

15 The coefficients on these country dummy variables are largest for Malawi and Nigeria, two countries with considerable input subsidy programs; however, we make no claim of a causal link between the existence of these schemes and these outcomes, particularly given mixed evidence on the performance of input subsidy programs across the region (e.g., Ricker-Gilbert et al., 2013).

16 When running this analysis with the same variables (apart from the fertilizer price) for agro-chemical use instead, the same qualitative patterns emerge. Country-level variables account for 43% of the variation, farm operation variables account for 20%, biophysical variables account for 15%, market and accessibility variables account for 15%, and socio-economic variables account for 7%. Holding constant other factors, both cash crops and maize production are negative and statistically significantly related to agro-chemical use, while the number of crops cultivated is positively associated with input use and accounts for 17% of the variation alone, suggesting that diversified producers may be more likely to use agro-chemicals across these six countries.
